# Epidemiological investigation of lower respiratory tract infections during influenza A (H1N1) pdm09 virus pandemic based on targeted next-generation sequencing

**DOI:** 10.3389/fcimb.2023.1303456

**Published:** 2023-12-14

**Authors:** Xiaodan Li, Yang Liu, Minzhe Li, Jing Bian, Demei Song, Chaoying Liu

**Affiliations:** ^1^ Department of Respiratory Medicine, The First Affiliated Hospital of Jilin University, Changchun, China; ^2^ Department of Respiratory and Critical Care Medicine, The First Hospital of Jilin University-the Eastern Division, Changchun, China

**Keywords:** influenza A (H1N1) pdm09 virus, pandemic, epidemiology, targeted next-generation sequencing, prognosis

## Abstract

**Background:**

Co-infection has been a significant contributor to morbidity and mortality in previous influenza pandemics. However, the current influenza A (H1N1) pdm09 virus pandemic, as the first major outbreak following the SARS-CoV-2 pandemic, may differ epidemiologically. Further investigation is necessary to understand the specific features and impact of this influenza A pandemic. Study design: We conducted a retrospective cohort study at a Chinese hospital between January and April 2023, focusing on patients with lower respiratory tract infections. Pathogen detection employed targeted next-generation sequencing (tNGS) on bronchoalveolar lavage fluid (BALF) or sputum samples.

**Results:**

This study enrolled 167 patients with lower respiratory tract infections, and the overall positivity rate detected through tNGS was around 80%. Among them, 40 patients had influenza A (H1N1) pdm09 virus infection, peaking in March. In these patients, 27.5% had sole infections, and 72.5% had co-infections, commonly with bacteria. The frequently detected pathogens were *Aspergillus fumigatus*, SARS-CoV-2, and *Streptococcus pneumoniae*. For non-influenza A virus-infected patients, the co-infection rate was 36.1%, with 42.3% having SARS-CoV-2. Patients with influenza A virus infection were younger, had more females and diabetes cases. Among them, those with sole infections were older, with less fever and asthma but more smoking history. Regarding prognosis, compared to sole influenza A virus infection, co-infected patients demonstrated higher 21-day recovery rates and a higher incidence of heart failure. However, they exhibited lower proportions of respiratory failure, acute kidney failure, septic shock, and hospital stays lasting more than 10 days. Interestingly, patients with non-influenza A virus infection had a significantly lower 21-day recovery rate. Correlation analysis indicated that the 21-day recovery rate was only associated with influenza A (H1N1) pdm09 virus.

**Conclusion:**

During the current pandemic, the influenza A (H1N1) pdm09 virus may have been influenced by the SARS-CoV-2 pandemic and did not exhibit a strong pathogenicity. In fact, patients infected with influenza A virus showed better prognoses compared to those infected with other pathogens. Additionally, tNGS demonstrated excellent detection performance in this study and showed great potential, prompting clinical physicians to consider its use as an auxiliary diagnostic tool.

## 1 Introduction

Influenza virus is a significant global health concern, responsible for a considerable burden of morbidity and mortality. As per the World Health Organization (WHO) data, influenza is associated with an annual death toll ranging from 0.29 to 0.65 million (Bal et al., 2020). In particular, the emergence of the influenza A (H1N1) pdm09 virus, which is of swine-origin, was first documented in the United States and Mexico in the year 2009 (CDC, 2009; Smith et al., 2009). Then it caused a widespread pandemic, affecting 214 countries (Zimmer and Burke, 2009). During this pandemic, a noteworthy observation was that 18-33% of patients developed bacterial infections alongside viral pneumonia (Estenssoro et al., 2010; Martín-Loeches et al., 2011; Nin et al., 2011). Studies have indicated that most influenza-related deaths occur due to secondary bacterial pneumonia rather than the virus itself (Brundage and Shanks, 2008; Morens et al., 2008; Chien et al., 2009).

The influenza pandemic underscored the importance of robust influenza surveillance and necessitated advancements in pathogen detection technologies. One such technology is targeted next-generation sequencing (tNGS), which utilizes probe hybridization capture or ultra-multiplex PCR amplification to enrich nucleic acid sequences of various pathogenic microorganisms. tNGS offers comprehensive coverage of common pathogens associated with influenza virus co-infections and boasts high sensitivity and specificity, making it a suitable tool for influenza virus monitoring (Gaston et al., 2022).

In August 2010, the WHO declared influenza A (H1N1) pdm09 virus as having evolved into a seasonal influenza virus, expected to circulate alongside other seasonal viruses (Cillóniz et al., 2012). However, in January 2023, the virus re-emerged in China, marking the first influenza pandemic after the SARS-CoV-2 pandemic. The severity of this pandemic is still uncertain. Consequently, this study aims to investigate the epidemiological and pathogenetic characteristics of influenza A (H1N1) pdm09 virus during this pandemic using tNGS and explore its impact on patient outcomes. By doing so, we aim to contribute valuable insights to the understanding of this novel influenza A (H1N1) pdm09 virus pandemic and its implications for public health.

## 2 Materials and methods

### 2.1 Study population and data collection

This retrospective, observational study focused on adult patients (age ≥ 18 years) hospitalized with a diagnosis of lower respiratory tract infections during the influenza A (H1N1) pdm09 virus pandemic. None of the individuals under scrutiny presented with positive clinical test results. The study enrolled patients from the First Bethune Hospital of Jilin University, covering the period from January to April 2023. The following information was recorded: demographic data, underlying medical conditions, infection symptoms, time from onset to admission, laboratory test results, duration of hospital stay, complications, and the recovery status within 21 days. To assess the severity of the disease, the researchers calculated the confusion-urea-respiratory rate-blood pressure-age 65 (CURB-65) score and as the sequential organ failure assessment (SOFA) score for all patients. Patients without complete clinical information were excluded from the analysis.

This study received approval from the Ethics Committees (Register: 2023-KS-130) to ensure compliance with ethical guidelines. Patients’ identification remained anonymous throughout the study, and informed consent was waived due to the retrospective and observational nature of the research and its significance as an emergency public health response.

### 2.2 Sample collection

Bronchoalveolar lavage fluid (BALF) or sputum samples were collected from eligible patients and stored in sterile screw-capped cryovials. It is important to note that the astute selection of BALF samples sourced from the middle segment, with the anterior segment’s collected fluid discarded. Similarly, preserved sputum samples were procured from patients’ first deep cough episodes in the early morning, following mouth rinsing with sterile saline 2-3 times. These samples were then transported to the designated laboratory for tNGS at ≤ - 20° to ensure sample integrity. For BALF samples, a volume of 5-10 mL was collected from each patient, while 4 mL of sputum was collected for each patient as well. These samples were carefully handled and preserved to maintain the quality of the genetic material for subsequent tNGS analysis.

### 2.3 Targeted next-generation sequencing

Sputum after liquefaction and BALF samples were collected and combined with lysis buffer, protease K mixture, and binding buffer in a grinding tube. The mixture was then disrupted using a shock breaker for 30 seconds. Subsequently, the lysate underwent simultaneous DNA and RNA extraction using the VAMNE Magnetic Pathogen DNA/RNA Extraction Kit (Vazyme, Nanjing, China). The quantification of nucleic acids was performed using a Qubit 3.0 fluorometer with double-stranded DNA (dsDNA) and RNA high-sensitivity (HS) reagents.

For cDNA synthesis and library preparation, we employed the HieffNGS^®^C37P4 One PotcDNA&gDNA Library Prep Kit (Yeasen, Shanghai, China) following the provided protocol. To enrich the target sequences, we incubated GenePlus probes with the samples for approximately 4 hours, followed by an 18-cycle PCR to amplify the captured products, which were subsequently prepared into DNA nanoballs (DNBs).

The sequencing procedure was carried out on the Gene^+^Seq-100 sequencing platform (GenePlus-Beijing) with 100-bp single-end read sequencing, aiming for a target depth of 5 million reads for the targeted workflow.

For data analysis, we utilized GenePlus’ self-built automated Data Analysis Solution. The raw data underwent preprocessing to remove low-quality sequences, residual adapters, and short reads. Additionally, microbial rRNA and human-derived sequences were excluded from further analysis. The filtered reads were aligned and annotated against the self-built pathogenic microorganism database using BLAST software. Reads aligned to the target capture interval of the probe for corresponding species were defined as target-reads and normalized as the number of reads per million data volume (target-RPMCR). The final output provided the sample’s pathogen list, aiding in the identification of suspected responsible pathogens.

### 2.4 Statistical analysis

Categorical variables were described by frequencies and percentages. Continuous variables were described by the median and interquartile range (IQR). Categorical variables were compared using the chi-square test. Continuous variables were compared using the Manne-Whitney U test. Univariate and multivariate logistic regression analyses were performed to identify variables predictive of patients’ recovery in 21 days. The choice of this dependent variable was considered by the clinical meaning, data availability, mortality rate, and follow-up period for most patients. While the independent variables analyzed included the infection of influenza A (H1N1) pdm09 virus, age, hypertension, diabetes, and smoking. All tests were two-tailed and significance was set at 5%. All figures were drawn using GraphPad Prism version 9.5.0 for Windows (GraphPad Software LLC., San Diego, CA, USA). All analyses were performed with SPSS version 26.0 for Windows (SPSS Inc., Chicago, Illinois, USA).

## 3 Results

**Figure 1:**
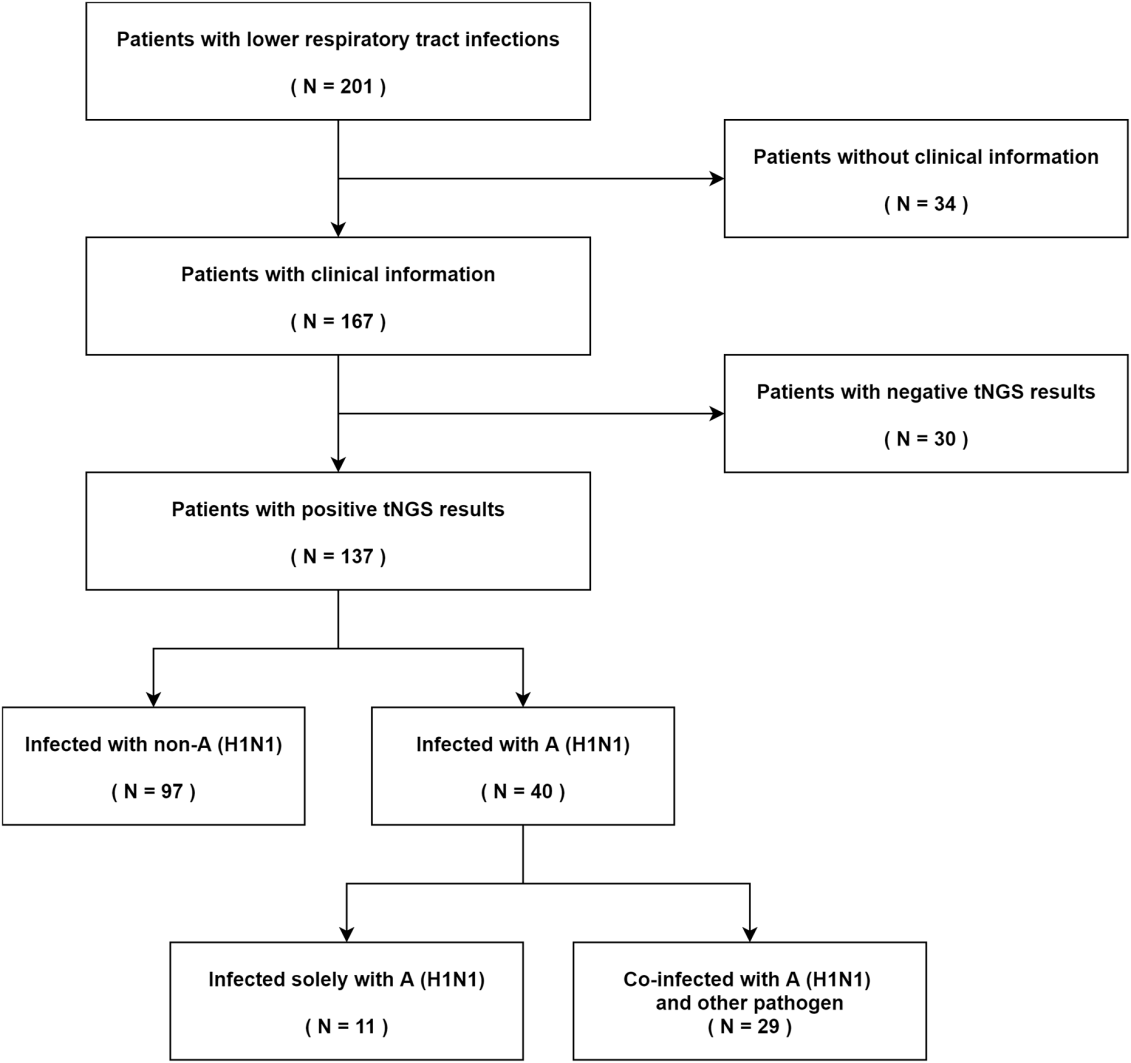
Screening algorithm of patients with lower respiratory tract infections.

During the influenza A (H1N1) pdm09 virus pandemic from January to April 2023 in China, a total of 201 patients with lower respiratory tract infections were initially tested. After the exclusion of 34 patients due to a lack of clinical information and 30 patients with negative test results, the study population consisted of 137 patients. tNGS was performed on these 137 patients, leading to the identification of positive results. Specifically, 11 patients were solely infected with the influenza A (H1N1) pdm09 virus, while 29 patients exhibited a co-infection of influenza A (H1N1) pdm09 virus along with other pathogens. Among the remaining 97 patients, the infection did not involve the influenza A (H1N1) pdm09 virus (**Figure 1**).

The main demographic and clinical characteristics of the study populations are detailed in **Table 1**. Patients infected with non-influenza A (H1N1) pdm09 virus demonstrated distinct demographic characteristics compared to those infected with influenza A (H1N1) pdm09 virus. Specifically, the former group was relatively older, had a higher proportion of males, and a lower proportion of diabetes. Among patients solely infected with influenza A (H1N1) pdm09 virus, several clinical differences were observed when compared to those co-infected. Patients with influenza A (H1N1) pdm09 virus sole infection were older, had a lower proportion of fever and asthma, and exhibited a lower prevalence of underlying conditions such as hypertension, diabetes, and chronic bronchitis. However, they presented with a higher proportion of individuals with a smoking history.

**Table 1:** Demographic and clinical characteristics.

Characteristics	Patients infected with non-influenza A (H1N1) pdm09 virus (N=97)	Patients infected with influenza A (H1N1) pdm09 virus			*P*-value	*P*-value’
		Total (N=40)	Single pathogen (N=11)	Multiple pathogens (N=29)		
Male, n (%)	63 (65)	22 (55)	6 (55)	16 (55)	0.275	0.972
Age (years), median (IQR)	66 (57-73)	61 (56-69)	66 (60-74)	60 (52-67)	0.273	0.127
Hypertension, n (%)	31 (32)	11 (28)	2 (18)	9 (31)	0.607	0.416
Diabetes, n (%)	18 (19)	12 (30)	2 (18)	10 (34)	0.141	0.315
Chronic bronchitis, n (%)	16 (16)	5 (13)	1 (9)	4 (14)	0.555	0.688
Smoking, n (%)	35 (36)	17 (43)	6 (55)	11 (38)	0.482	0.343
Cough, n (%)	69 (71)	34 (85)	9 (82)	25 (86)	0.088	0.729
Fever, n (%)	54 (56)	20 (50)	3 (27)	17 (59)	0.545	0.077
Asthma, n (%)	55 (57)	18 (45)	4 (36)	14 (48)	0.212	0.499
From onset to admission (days), median (IQR)	9 (5-20)	10 (6-15)	10 (7-13)	10 (5-15)	0.344	0.673

IQR, interquartile range. P-value, between patients with influenza A (H1N1) pdm09 virus infection and non-influenza A (H1N1) pdm09 virus infection; P-value’, between patients solely infected with influenza A (H1N1) pdm09 virus and co-infected with influenza A (H1N1) pdm09 virus and other pathogens.

As shown in **Table 2**, the main clinical examinations were no significant differences among the three groups. Compared to patients infected with influenza A (H1N1) pdm09 virus, patients infected with other pathogens exhibited lower white blood cell counts (*P* > 0.05), higher IL-6 concentrations (*P* > 0.05), and lower sequential organ failure assessment (SOFA) scores (*P* > 0.05). Among patients co-infected, they had higher levels of C-reactive protein (CRP) (*P* > 0.05) and lower SOFA scores (*P* > 0.05) when compared to patients solely infected with influenza A (H1N1) pdm09 virus.

**Table 2:** Clinical examination at admission.

Examination	Patients infected with non-influenza A (H1N1) pdm09 virus (N=97)	Patients infected with influenza A (H1N1) pdm09 virus			*P*-value	*P*-value’
		Total (N=40)	Single pathogen (N=11)	Multiple pathogens (N=29)		
Axillary temperature (°), median (IQR)	36.6 (36.5-38.5)	36.6 (36.5-38.3)	36.6 (36.5-38.0)	36.6 (36.5-38.4)	0.510	0.679
Respiratory rate (beats/min), median (IQR)	20 (20-22)	20 (20-24)	20 (20-23)	20 (20-24)	0.231	0.654
White blood cell count, median (IQR)	7.1 (5.3-9.6)	8.5 (5.5-11.6)	8.5 (3.7-10.8)	8.4 (6.1-11.6)	0.436	0.433
CRP (mg/L), median (IQR)	31.0 (7.1-89.0)	30.6 (14.4-104.3)	22.5 (14.3-41.8)	45.0(14.5-124.2)	0.597	0.238
PCT (μg/L), median (IQR)	0.081 (0.060-0.294)	0.095 (0.056-0.235)	0.067 (0.043-0.099)	0.112 (0.062-0.541)	0.820	0.185
IL-6 (ng/L), median (IQR)	33.6 (9.9-86.0)	18.3 (8.5-42.2)	13.9 (11.8-18.8)	20.4 (8.3-76.6)	0.193	0.533
CURB-65 score ≥ 2, n (%)	18 (19)	9 (23)	2 (18)	7 (24)	0.325	0.315
SOFA score ≥ 2, n (%)	41 (42)	21 (53)	8 (73)	17 (59)	0.096	0.630

CRP, C-reactive protein; PCT, procalcitonin; IL-6, interleukin- 6; CURB-65, confusion, urea, respiratory rate, blood pressure, age 65; SOFA, sequential organ failure assessment; P-value, between patients with influenza A (H1N1) pdm09 virus infection and non-influenza A (H1N1) pdm09 virus infection; P-value’, between patients solely infected with influenza A (H1N1) pdm09 virus and co-infected with influenza A (H1N1) pdm09 virus and other pathogens.

**Figure 2:**
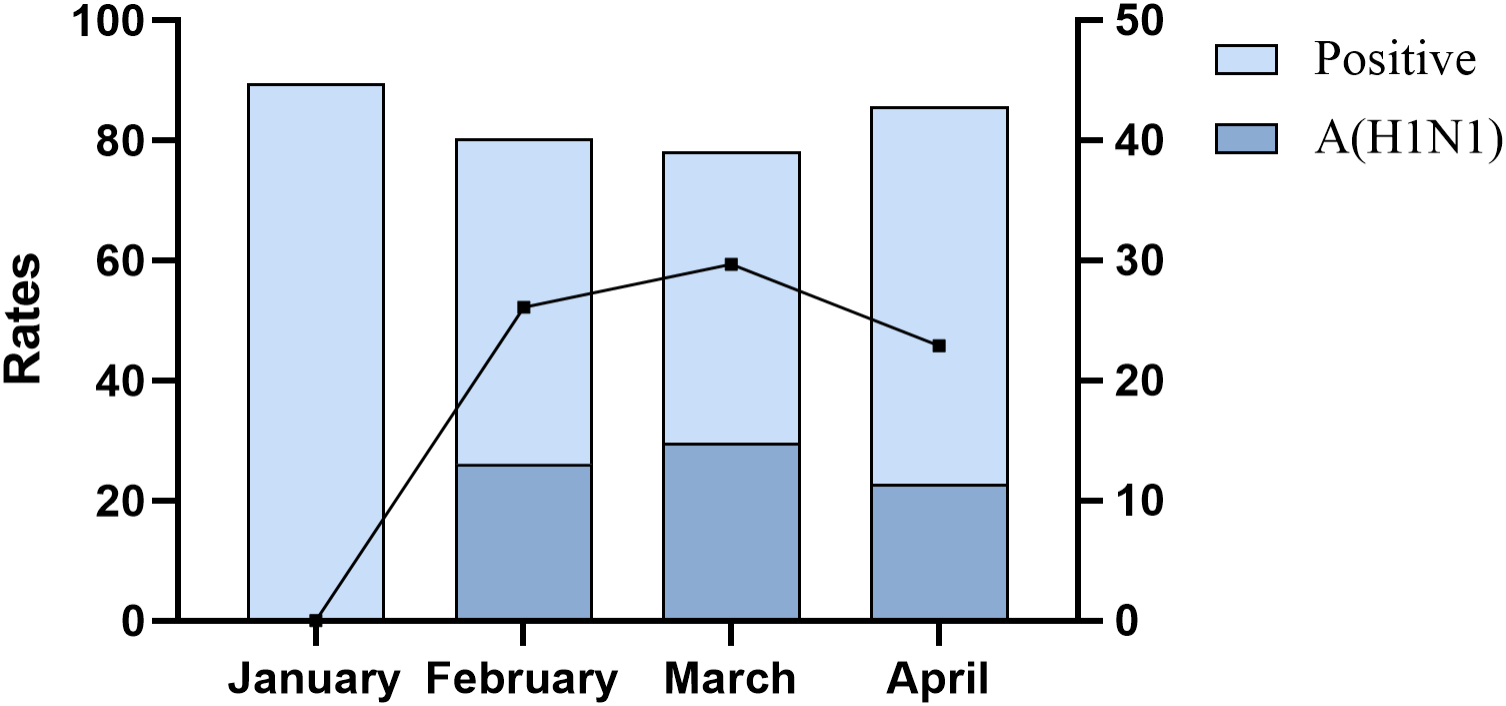
Positive rate and influenza A (H1N1) pdm09 virus positive rate of tNGS in patients with lower respiratory tract infections.

During the current influenza pandemic, the positivity rate of tNGS among patients with lower respiratory tract infections remained relatively stable, hovering around 80% from January to April. The detection of influenza A (H1N1) pdm09 virus was predominantly observed in February (32.4%), March (38.0%), and April (26.7%), with March showing the highest positivity rate during this period (**Figure 2**).

**Figure 3:**
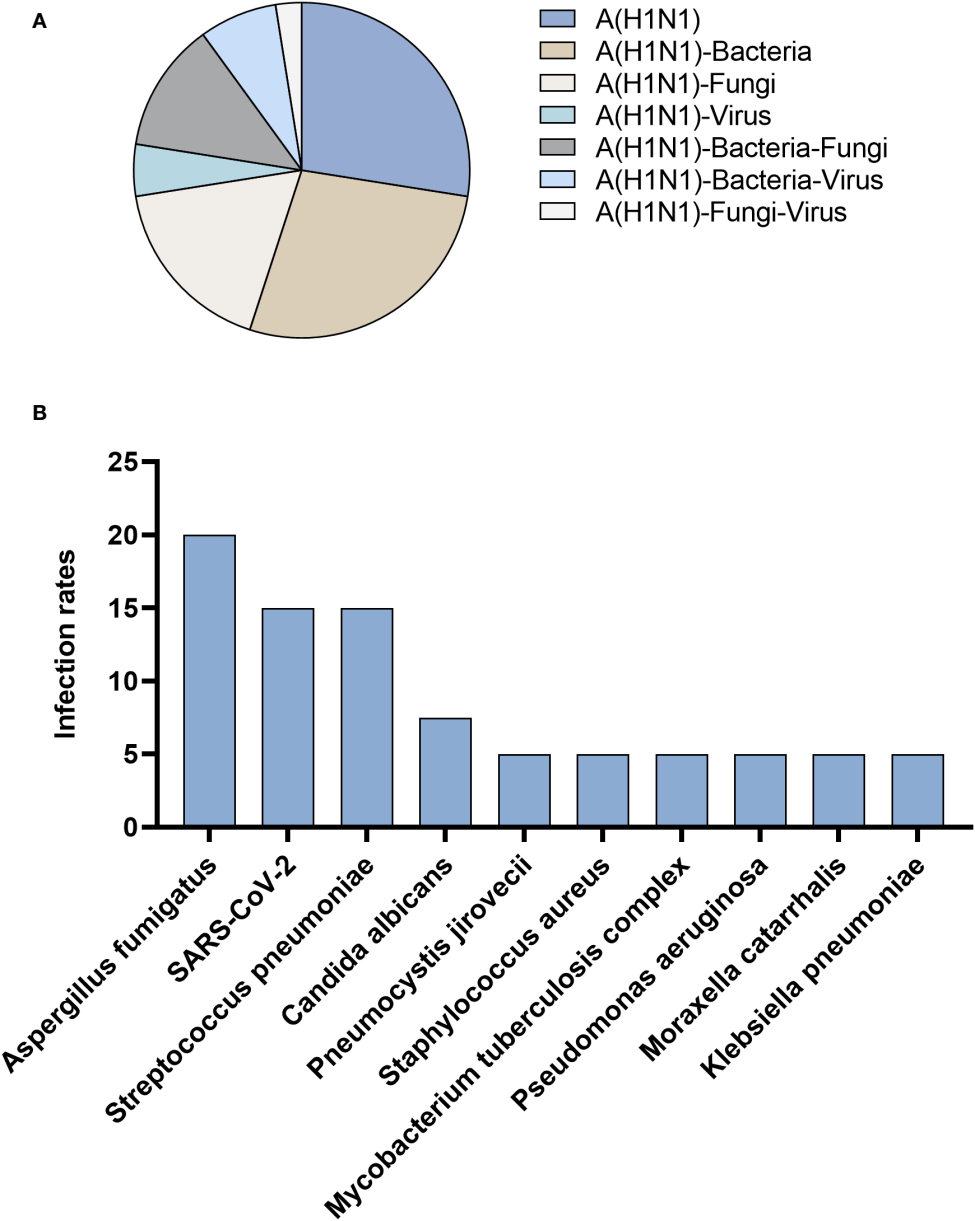
Patients infected with influenza A (H1N1) pdm09 virus. **(A)** Distribution of infection types. **(B)** Distribution of infection pathogens.

Among 40 patients infected with influenza A (H1N1) pdm09 virus, 27.5% had influenza A (H1N1) pdm09 virus sole infection, and 72.5% had co-infection. The most prevalent types of co-infections included combinations with bacteria (27.5%), fungi (17.5%), and both bacteria and fungi (12.5%) (**Figure 3A**). In addition to the influenza A (H1N1) pdm09 virus, the most frequently detected pathogens by tNGS were *Aspergillus fumigatus* (20.0%), followed by SARS-CoV-2 (15.0%) and *Streptococcus pneumoniae* (15.0%) (**Figure 3B**).

**Figure 4:**
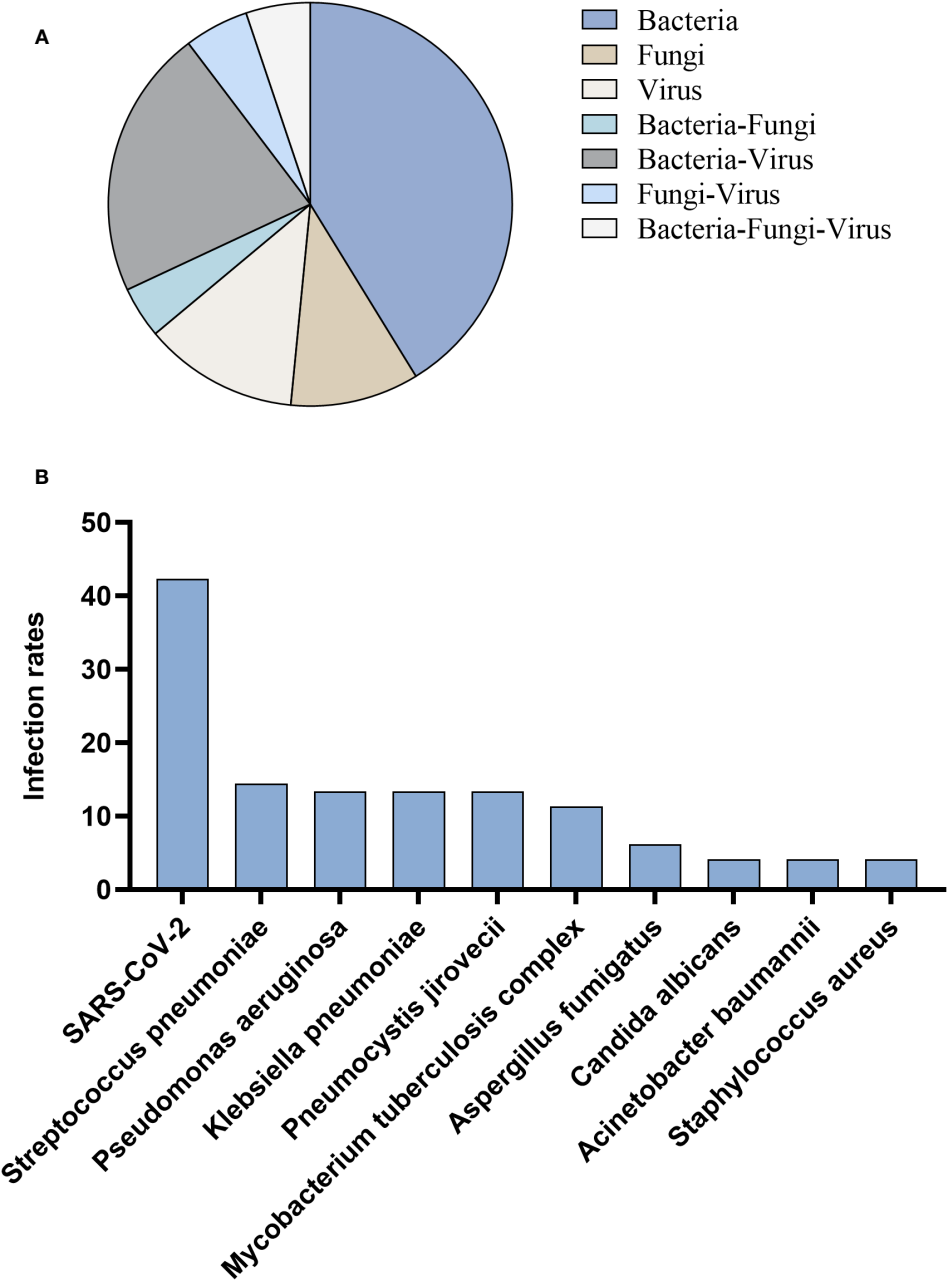
Patients infected with non-influenza A (H1N1) pdm09 virus. **(A)** Distribution of infection types. **(B)** Distribution of infection pathogens.

Among 97 patients infected with non-influenza A (H1N1) pdm09 virus, the most prevalent type of infection was sole infection, accounting for 63.9%, while co-infection accounted for 36.1%. Co-infection cases primarily involved a combination of bacteria and virus (21.6%) (**Figure 4A**). In the context of co-infection, tNGS identified SARS-CoV-2 (42.3%) as the most frequently detected pathogen, followed by *Streptococcus pneumoniae* (14.4%), *Pseudomonas aeruginosa* (13.4%), *Klebsiella pneumoniae* (13.4%), *Pneumocystis jirovecii* (13.4%), *Mycobacterium tuberculosis* complex (11.3%) (**Figure 4B**).

**Figure 5:**
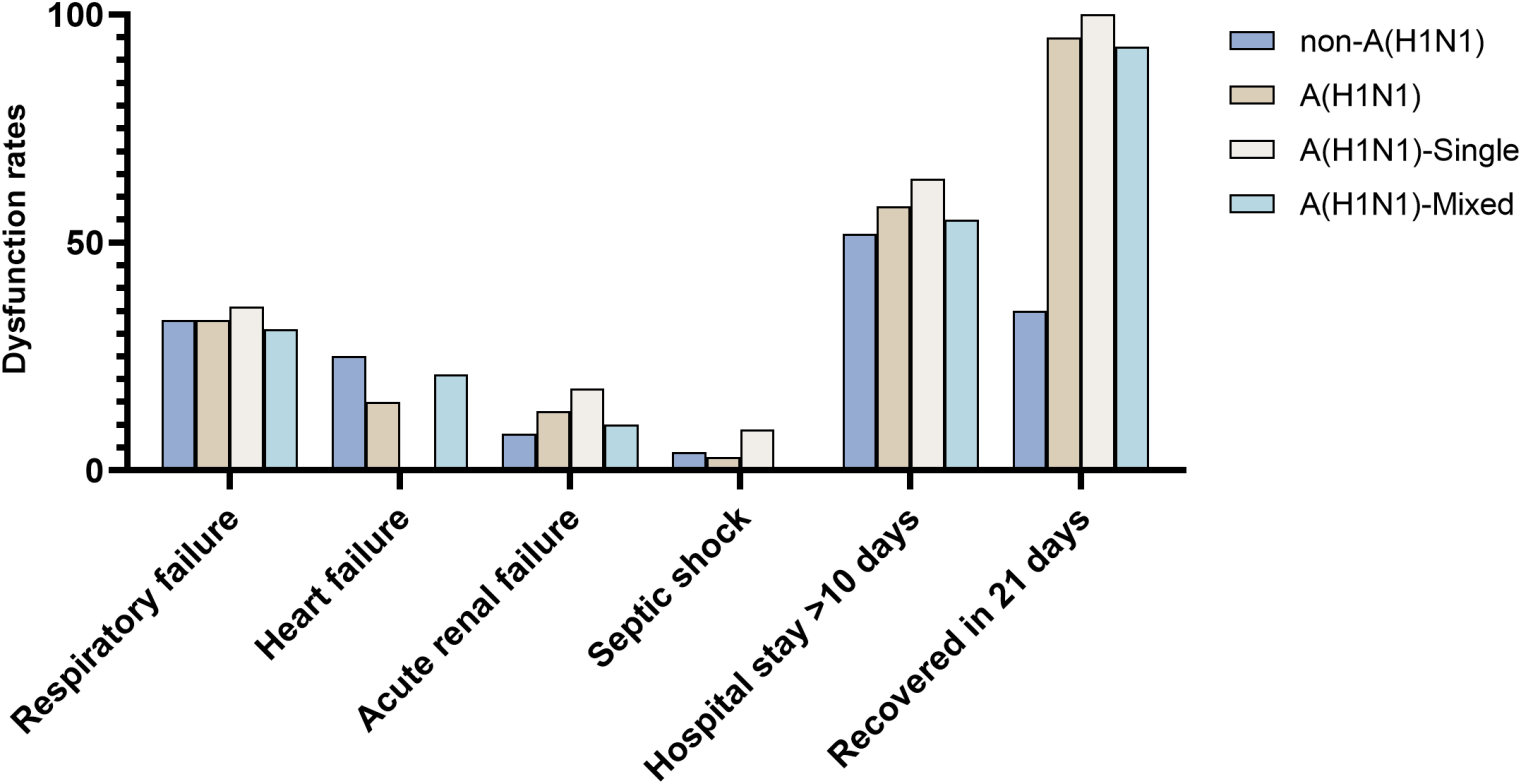
Clinical prognosis and outcomes.

In terms of clinical prognosis and outcomes, respiratory failure was the most common complication, with an incidence rate of over 30% in all patient groups. Heart failure had a higher occurrence rate in patients with non-influenza A (H1N1) pdm09 virus infection and in patients with influenza A (H1N1) pdm09 virus co-infection, at 25% and 21%, respectively. However, it did not occur in patients with influenza A (H1N1) pdm09 virus sole infection. Acute renal failure occurred more frequently in patients with influenza A (H1N1) pdm09 virus infection (13% vs. 8%), particularly in those with sole infection (18% vs. 10%). Septic shock had the highest incidence rate in patients with influenza A (H1N1) pdm09 virus sole infection (9%), followed by patients with non-influenza A (H1N1) pdm09 virus infection (4%), and it did not occur in patients with influenza A (H1N1) pdm09 virus co-infection. Furthermore, approximately 60% of patients had a hospital stay longer than 10 days, with the highest rate observed in patients with influenza A (H1N1) pdm09 virus sole infection (64%). Notably, there was a significant difference in the 21-day recovery rate between patients with influenza A (H1N1) pdm09 virus infection and those with non-influenza A (H1N1) pdm09 virus infection (95% vs 35%). All patients with influenza A (H1N1) pdm09 virus sole infection recovered within 21 days, indicating a faster recovery in this group (**Figure 5**).

The results of the risk factor analysis are presented in **Table 3**. Both the univariate and multivariate logistic regression models indicate that patients’ recovery within 21 days was only associated with the infection of influenza A (H1N1) pdm09 virus. Factors such as age, hypertension, diabetes, and smoking did not have a significant influence on the 21-day recovery outcome, as observed in both regression models.

**Table 3:** Logistic regression analysis of risk factors for patients’ recovery in 21 days.

Variable	Univariate logistic analysis		Multivariate logistic analysis	
	*OR* (95% *CI*)	*P*-value	*OR* (95% *CI*)	*P*-value
A (H1N1)	0.030 (0.000-0.100)	<0.001	0.030 (0.000-0.100)	<0.001
Age	0.990 (0.970-1.020)	0.632	1.000 (0.970-1.030)	0.980
Hypertension	1.140 (0.550-2.370)	0.731	1.370 (0.570-3.270)	0.480
Diabetes	1.470 (0.650-3.420)	0.357	1.060 (0.360-2.980)	0.915
Smoking	1.230 (0.620-2.480)	0.556	1.110 (0.470-2.570)	0.811

OR, Odd ratio; CI, Confidence interval.

## 4 Discussion

Following the SARS-CoV-2 pandemic, the influenza pandemic was the first major outbreak to occur in early 2023. Prior to our study, there were no research findings to indicate whether influenza pandemics occurring after the SARS-CoV-2 pandemic exhibited any differences from previous seasonal influenza outbreaks, especially regarding its severity. To address this knowledge gap, we conducted this study with the aim of investigating this issue. Our study’s findings indicate a noteworthy decrease in the pathogenicity of the influenza A (H1N1) pdm09 virus during the current pandemic. The observed phenomenon is characterized by a diminished prevalence of lower respiratory tract infections attributed to the influenza A (H1N1) pdm09 virus during the pandemic period, indicating a predilection for upper respiratory tract infections. Concurrently, a salient feature is the improved clinical prognosis discerned among patients afflicted with lower respiratory tract infections caused by the influenza A (H1N1) pdm09 virus. It is evident that the current pandemic of influenza A (H1N1) pdm09 virus has been influenced to some extent by the preceding SARS-CoV-2 pandemic. This observation sheds light on the complex interplay between different viral outbreaks and raises important considerations for future pandemic preparedness and response strategies. Further research and continuous monitoring are essential to comprehensively understand the dynamic interactions between respiratory viruses and their impact on public health.

In the period from January to April 2023, our study collected a total of 167 samples with comprehensive patient clinical information. The overall positivity rate of pathogen detection in these samples was approximately 80%, showcasing the remarkable detection performance of tNGS. In a previous study (Gaston et al., 2022), a comparison between tNGS and mNGS found that both methods had similar pathogen detection performance, with tNGS sensitivity at 45.9%. This sensitivity is notably lower than what we observed in our actual clinical testing. Although the tNGS used in the two studies may not be the same product, it still suggests that tNGS technology has the potential for highly efficient pathogen detection. Notably, despite using tNGS in our study, we did not observe significant differences in clinical characteristics and examination results among the patient groups. This finding indirectly points to the challenge in clinically differentiating between single pathogen infections and mixed infections. Consequently, we strongly recommend clinicians to adopt accurate and rapid diagnostic methods such as tNGS to improve the diagnostic success rate when identifying infections. Furthermore, extant literature underscores that we must prepare now for seasonal and pandemic influenza. Effective preparedness necessitates vigilant pathogen surveillance, facilitated by molecular assays characterized by expeditious turnaround times, heightened sensitivity, and impeccable accuracy (Dzau and Yadav, 2023).

In our study, we observed that the peak month of the influenza pandemic was March, which was later than the findings reported in previous studies (Chiarella et al., 2017; Pando et al., 2017). We speculate that this delay might be influenced by the later stages of the SARS-CoV-2 pandemic, but further research is necessary to confirm this hypothesis. Among the 40 patients infected with the influenza A (H1N1) pdm09 virus, 29 cases (72.5%) were detected with co-infections of other pathogens through tNGS. Among these cases, co-infections with bacteria accounted for 11 cases (27.5%), which is consistent with the occurrence rate of bacterial-viral co-infections reported in previous studies, ranging from 18% to 33% (Estenssoro et al., 2010; Martín-Loeches et al., 2011; Nin et al., 2011). In the 97 cases of non-influenza A (H1N1) pdm09 virus infections, the rate of co-infections was also relatively high, with 35 cases (36.1%) identified. The majority of co-infections were with SARS-CoV-2, accounting for 42.3% of all non-influenza A (H1N1) pdm09 virus infections. The high rate of co-infections with SARS-CoV-2 has been confirmed in previous studies and aligns with our findings (Hoque et al., 2021; Alhumaid et al., 2022). Interestingly, we also discovered that SARS-CoV-2 was the most frequently detected virus among patients infected with influenza A (H1N1) pdm09 virus, accounting for 15% of the cases. This indicates that the impact of the SARS-CoV-2 pandemic may not have completely subsided, but has become a regular occurrence (Davis et al., 2023; Liew et al., 2023). Additionally, among patients infected with influenza A (H1N1) pdm09 virus, *Aspergillus fumigatus* was the most frequently identified co-infecting pathogen, detected in 20% of the samples. This finding might be somewhat surprising, but previous research has found that some influenza patients may have concurrent invasive pulmonary aspergillosis (IPA), which may be related to the patients’ immune status (Huang et al., 2019; Waldeck et al., 2020). In our study, out of the 8 cases of influenza A (H1N1) pdm09 virus and *Aspergillus fumigatus* co-infections, 7 cases had underlying conditions such as hypertension, diabetes, and chronic bronchitis, indicating immunocompromised states. These results highlight the importance of considering patients’ immune status and underlying health conditions in the context of co-infections during influenza outbreaks. Further investigation is warranted to better understand the implications of such co-infections on patient outcomes and management strategies.

According to previous research, the severity of symptoms in patients with influenza virus co-infections does not appear to be more severe than those with sole infections (Pérez-García et al., 2016; Pando et al., 2017). This aligns with the results of our study. In our investigation, patients with co-infections, compared to those with influenza A (H1N1) pdm09 virus sole infection, exhibited higher 21-day recovery rates and higher rates of heart failure. However, they had lower proportions of respiratory failure, acute kidney failure, septic shock, and hospital stays lasting more than 10 days. In the risk factor analysis, whether in univariate analysis or multivariate analysis, the 21-day recovery rate was only associated with influenza A (H1N1) pdm09 virus infection. Interestingly, the 21-day recovery rate of patients with influenza A (H1N1) pdm09 virus infection was significantly higher than that of patients with non-influenza A (H1N1) pdm09 virus infections, indicating a markedly better prognosis for patients infected with influenza A (H1N1) pdm09 virus compared to other pathogens. This finding is in contrast to some previous research (Chaves et al., 2013; Töpfer et al., 2014; Rozencwajg et al., 2018; Lytras et al., 2020). In prior studies conducted within Indian ICU, it has been observed that the 28-day mortality rate among patients afflicted with influenza A (H1N1) virus infection reached a considerable incidence, with reported figures as elevated as 20.1% (Golagana et al., 2023). We speculate that this disparity may be attributed to a complex evolutionary process that influenza A (H1N1) pdm09 virus underwent during the 3-year period of the SARS-CoV-2 pandemic, potentially leading to a weakening of its pathogenicity. However, it is crucial to acknowledge that further research is warranted to confirm and fully comprehend the underlying reasons behind this unexpected outcome. Understanding the changes in pathogenicity and clinical outcomes of influenza A (H1N1) pdm09 virus during and after the SARS-CoV-2 pandemic could have significant implications for public health strategies and preparedness in managing future influenza outbreaks.

In conclusion, during this pandemic of influenza A (H1N1) pdm09 virus reveals a higher occurrence of co-infections likely influenced by the residual impact of the preceding SARS-CoV-2 pandemic. Importantly, patients infected with influenza A (H1N1) pdm09 virus demonstrated more favorable prognoses compared to infections with other pathogens. Notably, the recovery rate was found to be specifically correlated with influenza A (H1N1) pdm09 virus infection. Furthermore, our study underscores the significance of tNGS as a rapid and accurate detection method that can effectively aid in clinical diagnosis. Given its superior detection performance, we highly recommend its integration into routine testing practices. The findings from this research contribute to the understanding of the dynamics of respiratory viral outbreaks, especially in the context of co-infections, and they emphasize the importance of continuous monitoring and preparedness for future pandemic scenarios. As the interplay between different viral outbreaks remains complex, ongoing research is crucial to comprehensively grasp their impact on public health and to devise effective strategies for diagnosis, management, and response.

## Data availability statement

The data presented in the study are deposited in the China National Center for Bioinformation - National Genomics Data Center - Genome Sequence Archive, submission number subCRA021674.

## Ethics statement

The study design received approval from the Ethics Committee of the First Bethune Hospital of Jilin University (2023-KS-130).

## Author contributions

XL: Conceptualization, Data curation, Writing – original draft. YL: Conceptualization, Writing – original draft. ML: Data curation, Writing – original draft. JB: Data curation, Writing – original draft. DS: Visualization, Writing – original draft. CL: Writing – review & editing, Conceptualization.
